# Supplementary material for: Substantial evidence against sex differences in bed nucleus of the stria terminalis volume in the human brain
Source: Imaging Neurosci (Camb). 2026 Oct 27;4:IMAG.a.1420. doi: 10.1162/IMAG.a.1420 (PMC13716282; doi:10.1162/IMAG.a.1420)
Supplement: Supplementary file 1 [file IMAG.a.1420_supp.pdf]

Supplementary Material

Supplementary Table 1: Right BNST Bayesian Regression Results

Fixed effects

Predictor	Estimate	95% Credible Interval
Intercept	-0.04	-0.26 to 0.20
Sex (dummy)	0.17	-0.25 to 0.61
Age (Z)	-0.09	-0.28 to 0.09
Age^2 (Z)	0.05	-0.10 to 0.20
TBV (Z)	0.25	0.05 to 0.45

Random effects (variance components)

Component	Variance
Family	0.11
MZ pair	0.21
Residual	0.52

Model fit

Metric	Value
ICC (family only)	0.13
ICC (family + MZ)	0.37
Marginal R^2	0.16
Conditional R^2	0.50
Observations	170

Supplementary Table 2: Left BNST Bayesian Regression Results

Fixed effects

Predictor	Estimate	95% Credible Interval
Intercept	-0.06	-0.28 to 0.16
Sex (dummy)	0.16	-0.26 to 0.58
Age (Z)	-0.10	-0.28 to 0.08
Age^2 (Z)	0.07	-0.07 to 0.21
TBV (Z)	0.28	0.09 to 0.48

Random effects (variance components)

Component	Variance
Family	0.07
MZ pair	0.24
Residual	0.50

Model fit

Metric	Value
ICC (family only)	0.09
ICC (family + MZ)	0.38
Marginal R^2	0.18
Conditional R^2	0.52
Observations	170

Supplementary Table 1: Sample characteristics by reported sex. Values are mean (SD) [range] for age and mean (SD) for volumes.

Measure	Total (N = 170)	Female (n = 105)	Male (n = 65)
Age, years	29.44 (3.25) [22-36]	30.51 (2.60) [23-36]	27.69 (3.45) [22-34]
Total brain volume, mm^3	1,166,579.6 (115,090.6)	1,106,531.3 (79,382.5)	1,263,580.9 (96,256.0)
Bilateral BNST volume, mm^3	113.73 (25.05)	107.72 (24.70)	123.45 (22.59)
Left BNST volume, mm^3	57.79 (13.52)	54.56 (13.05)	63.01 (12.68)
Right BNST volume, mm^3	55.94 (12.26)	53.16 (12.40)	60.44 (10.65)

Supplementary Table 2: Family structure by reported sex.

Family structure	Total	Female	Male
Monozygotic twin-pair participants	80	58	22
Dizygotic twin-pair participants	72	38	34
Non-twin sibling participants	2	1	1
Unrelated participants	16	8	8
Total participants	170	105	65
